# Supplementary material for: Seasonal changes in the structure of river fish communities in temperate Japan depicted using quantitative eDNA metabarcoding
Source: PLoS One. 2025 Jul 16;20(7):e0328280. doi: 10.1371/journal.pone.0328280 (PMC12266392; doi:10.1371/journal.pone.0328280)
Supplement: S1 Table — (DOCX) [file pone.0328280.s001.docx]

| Sampling station | longitude | Latitude | Distance from the river mouth (km) | Elevation (m) | River width (m) |
| --- | --- | --- | --- | --- | --- |
| St. 1 | 35°27'17.1"N | 135°19'44.2"E | 0.6 | 5 | 86.5 |
| St. 2 | 35°26'53.4"N | 135°20'11.1"E | 1.6 | 7 | 20.5 |
| St. 3 | 35°26'11.3"N | 135°20'06.7"E | 2.89 | 9 | 32 |
| St. 4 | 35°25'31.3"N | 135°20'05.9"E | 4.07 | 10 | 4.6 |
| St. 5 | 35°25'31.6"N | 135°20'03.4"E | 4.07 | 10 | 4.5 |
| St. 6 | 35°24'31.1"N | 135°21'30.6"E | 7.64 | 39 | 4.4 |
| St. 7 | 35°24'52.9"N | 135°22'51.5"E | 10.85 | 109 | 28.5 |
| St. 8 | 35°24'49.9"N | 135°22'53.2"E | 10.93 | 111 | 1.7 |
| St. 9 | 35°23'45.8"N | 135°19'07.8"E | 7.87 | 35 | 5.4 |
| St. 10 | 35°22'01.8"N | 135°19'48.3"E | 13.1 | 85 | 7 |
| St. 11 | 35°22'01.5"N | 135°19'48.7"E | 13.11 | 86 | 15.5 |
| St. 12 | 35°23'02.5"N | 135°21'59.3"E | 17.79 | 213 | 1.7 |

**S1 Table.** Information on sampling location.

Sampling dates were 5 and 19 May 2023 (spring), 2 Aug. 2023 (summer), 9 Nov. 2023 (fall), and 16 Feb. 2024 (winter). Note that sampling at Sts. 3 and 12 in the spring were conducted on 19 May 2023, and the other sites in spring were on 5 May 2023.
