# Supplementary material for: Seasonal changes in the structure of river fish communities in temperate Japan depicted using quantitative eDNA metabarcoding
Source: PLoS One. 2025 Jul 16;20(7):e0328280. doi: 10.1371/journal.pone.0328280 (PMC12266392; doi:10.1371/journal.pone.0328280)
Supplement: S5 Fig — (a) Spring, (b) summer, (c) fall, (d) winter, and (e) all season. The distance indicates distance from the river mouth. In all season, sp, su, fa, and wi indicate spring, summer, fall, and winter, respectively. (PDF) [file pone.0328280.s005.pdf]

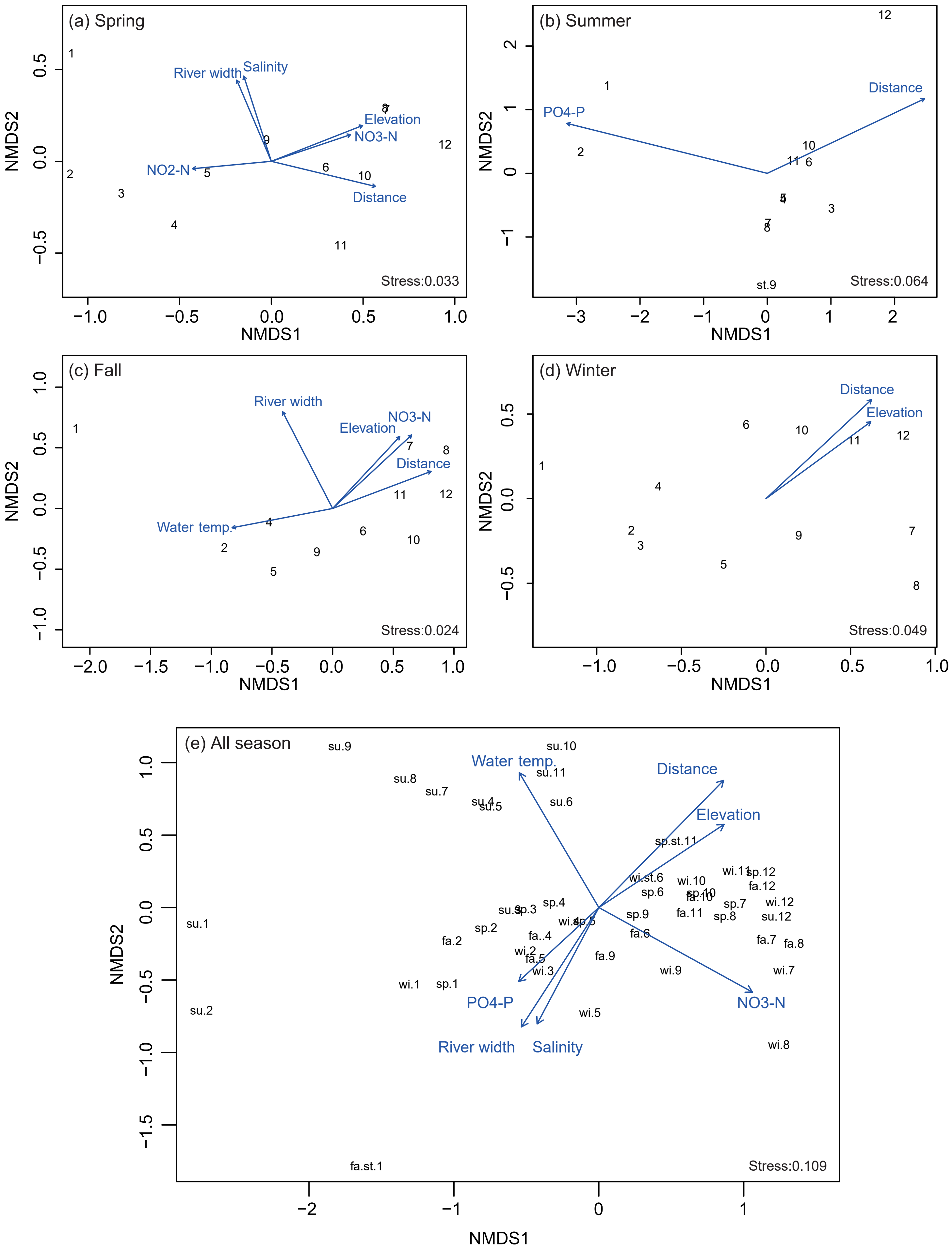

S5 Fig. NMDS plot with vectors of environmental factors and underwater materials. (a) spring, (b) summer, (c) fall, (d) winter, and (e) all season. The distance indicates distance from the river mouth. In all season, sp, su, fa, and wi indicate spring, summer, fall, and winter, respectively.
