# Supplementary material for: Seasonal changes in the structure of river fish communities in temperate Japan depicted using quantitative eDNA metabarcoding
Source: PLoS One. 2025 Jul 16;20(7):e0328280. doi: 10.1371/journal.pone.0328280 (PMC12266392; doi:10.1371/journal.pone.0328280)
Supplement: S6 Fig — (a) NO2-N, (b) NO3-N, and (c) PO4-P. The left panels indicate the difference of these concentrations among season, and the right panels show the relationship between the distance and these concentrations. (PDF) [file pone.0328280.s006.pdf]

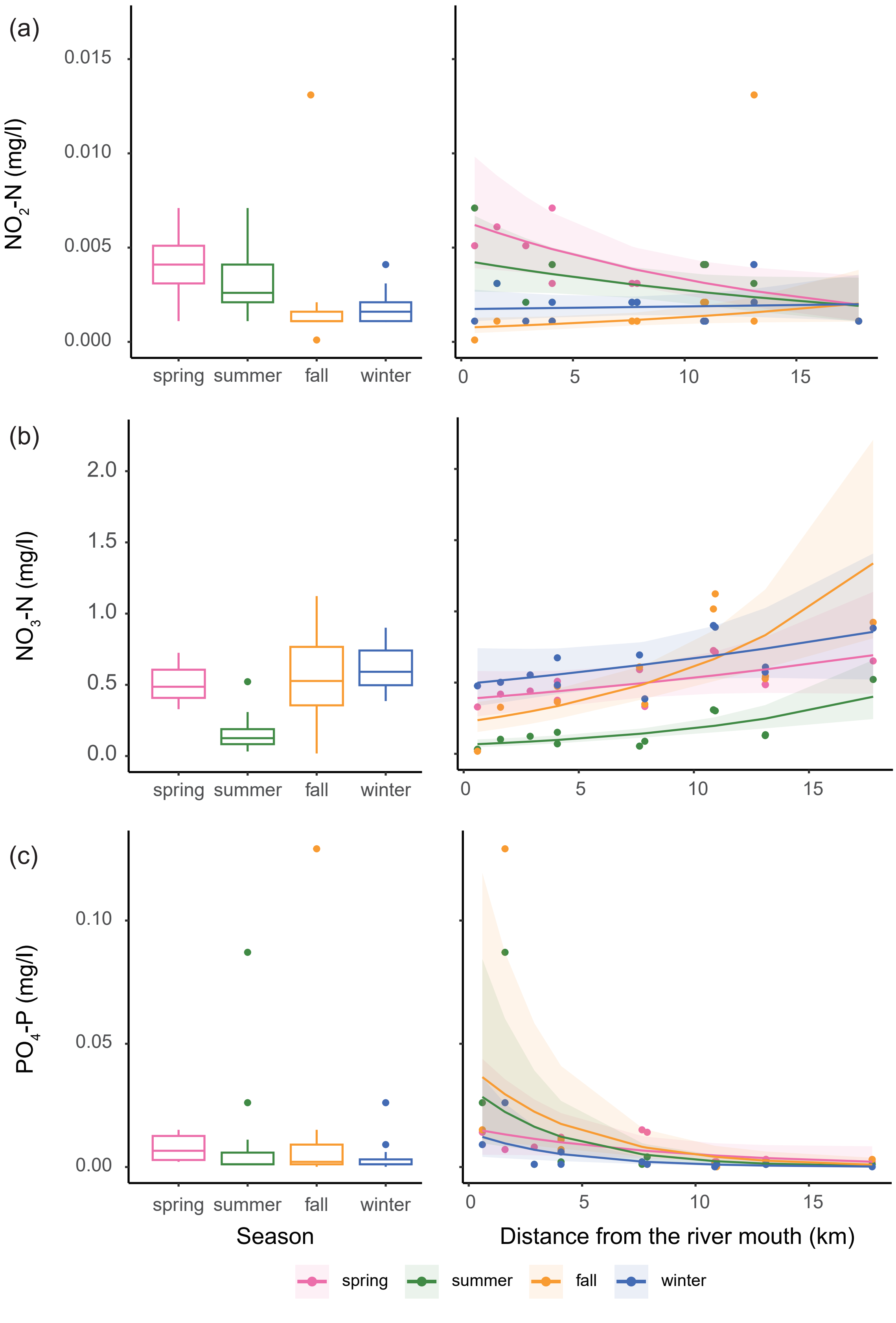

S6 Fig. The relationship between the distance from the river mouth and the concentration of underwater materials in different seasons. (a) NO_2-N, (b) NO_3-N, and (c) PO_4-P. The left panels indicate the difference of these concentrations among season, and the right panels show the relationship between the distance and these concentrations.
